# Development, Optimization, and Clinical Relevance of Lactoferrin Delivery Systems: A Focus on Ocular Delivery

**DOI:** 10.3390/pharmaceutics16060804

**Published:** 2024-06-14

**Authors:** Erika Ponzini, Gloria Astolfi, Rita Grandori, Silvia Tavazzi, Piera Versura

**Affiliations:** 1 Department of Materials Science, University of Milano-Bicocca, via R. Cozzi 55, I-20125 Milan, Italy; 2 COMiB Research Center, University of Milano-Bicocca, via R. Cozzi 55, I-20125 Milan, Italy; 3 Ophthalmology Unit, Dipartimento di Scienze Mediche e Chirurgiche (DIMEC), Alma Mater Studiorum Università di Bologna, via Palagi 9, I-40138 Bologna, Italy; gloria.astolfi2@unibo.it (G.A.); piera.versura@unibo.it (P.V.); 4 Department of Biotechnology and Biosciences, University of Milano-Bicocca, Piazza della Scienza 2, I-20126 Milan, Italy; rita.grandori@unimib.it; 5 Institute for Advanced Simulations, Forschungszentrum Juelich, 52428 Juelich, Germany; 6 IRCCS Azienda Ospedaliero-Universitaria di Bologna, via Palagi 9, I-40138 Bologna, Italy

**Keywords:** lactoferrin detection method, lactoferrin delivery strategies, ocular surface system, ocular posterior segment disease, topical ophthalmic administration, protein drugs, topical ocular administration

## Abstract

Lactoferrin (Lf), a multifunctional protein found abundantly in secretions, including tears, plays a crucial role in ocular health through its antimicrobial, immunoregulatory, anti-inflammatory, and antioxidant activities. Advanced delivery systems are desirable to fully leverage its therapeutic potential in treating ocular diseases. The process of Lf quantification for diagnostic purposes underscores the importance of developing reliable, cost-effective detection methods, ranging from conventional techniques to advanced nano-based sensors. Despite the ease and non-invasiveness of topical administration for ocular surface diseases, challenges such as rapid drug elimination necessitate innovations, such as Lf-loaded contact lenses and biodegradable polymeric nanocapsules, to enhance drug stability and bioavailability. Furthermore, overcoming ocular barriers for the treatment of posterior segment disease calls for nano-formulations. The scope of this review is to underline the advancements in nanotechnology-based Lf delivery methods, emphasizing the pivotal role of multidisciplinary approaches and cross-field strategies in improving ocular drug delivery and achieving better therapeutic outcomes for a wide spectrum of eye conditions.

## 1. Lactoferrin Biological Functions

Lactoferrin, also known as lactotransferrin (Lf), is a mammalian, first-line defense, multifunctional protein belonging to the transferrin family, which displays the specific ability to bind ferric iron. It is constitutively expressed in mucosal surfaces and secretory fluids, primarily milk, but also in the digestive tract, bile, saliva, bronchial and nasal secretion, cervicovaginal mucus, seminal fluids, and tears [1,2]. In addition, Lf is promptly delivered by circulating neutrophils to sites of microbial invasion [3].

**Figure 1:**
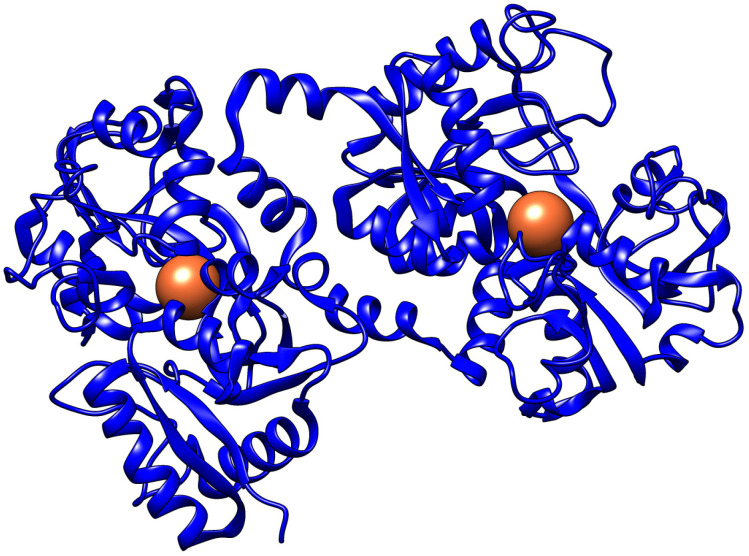
Structure of diferric human lactoferrin [5]. Fe^3+^ ions are highlighted as 3D spheres.

Lf is a globular cationic glycoprotein with a molecular mass of about 80 kDa consisting of two homologous domains known as the N-terminal and C-terminal lobes, with each lobe binding a single Fe^3+^ ion [4] (Figure 1).

**Figure 2:**
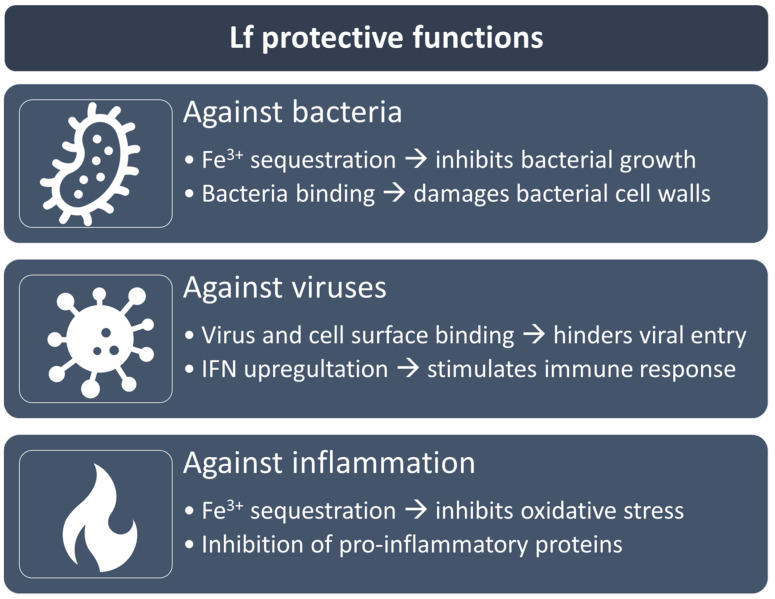
Schematic representation of the main protective functions of Lf. Abbreviations: Lf: lactoferrin; IFN: interferon.

Lf is known as an important antibacterial protein. Thanks to its iron-chelating ability (Figure 2), it sequesters ferric ions required for bacterial growth and prevents the formation of iron-dependent hydroxyl radicals by microbial infections [3], with different efficacy depending on the type of microorganisms [4,6].

Immunoregulatory functions of Lf are associated with its cationic charge, favoring interactions with negatively charged immune cells, hence modulating several cellular processes including differentiation, migration, and proliferation.

In addition, Lf displays anti-inflammatory, anti-cancer, and antioxidant activities, among several other physiological functions [7] (Figure 2). Applications of Lf in various anatomical districts have been comprehensively detailed in recent reviews [1,4,8,9,10,11,12,13].

A specific focus is devoted here to the role played by Lf in the ocular surface system (OSS) and within the eye. In particular, the aim of this manuscript is to review methods for detecting and delivering Lf for ocular treatments.

**Figure 3:**
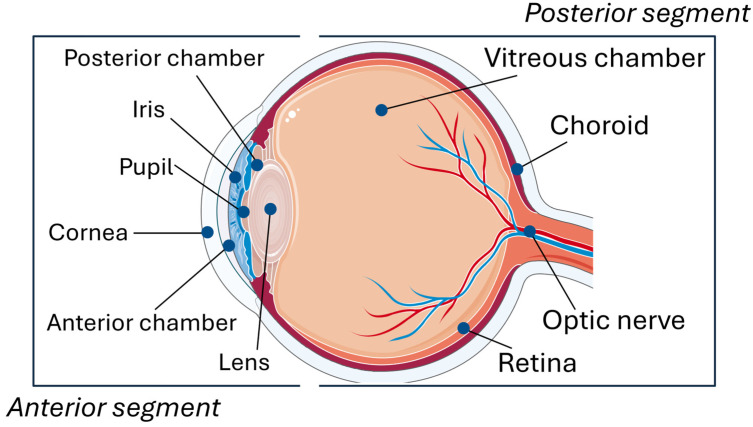
Schematic representation of the eye.

The eyeball can be divided into two distinct sections, an anterior and a posterior segment (Figure 3). The anterior segment encompasses the cornea, iris, ciliary body, lens, and the spaces known as the anterior and posterior chambers, which are filled with aqueous humor. The posterior segment is made up of the retina, choroid, optic nerve head, vitreous compartment, retinal pigment epithelium (RPE), and blood–retina barrier (BRB). The OSS is a morpho-functional unit, which is part of the anterior segment, and includes components functionally linked by the continuity of the epithelia, innervation and endocrine, and vascular and immune systems [14]. These components are the epithelia of the cornea and conjunctiva, tear film, eyelids, and lacrimal and meibomian glands.

Innate defenses of OSS comprise integrated anatomical, mechanical, and immunological mechanisms [15], with tear fluid playing a key defensive role as it contains several protective molecules [16,17,18]. Indeed, proteins, glycoproteins, and lipids contained in tears are key factors in maintaining a well-lubricated and smooth optical surface [17,19,20]. While the role of tear Lf against bacterial growth and biofilm formation is well established [6,21], evidence of efficacy in the setting of viral infections has emerged recently [22]. It is well known, for example, that oral delivery of Lf has proved useful in the prevention and treatment of COVID-19, with significant reductions in D-dimer, interleukin-6 (IL-6), and ferritin levels [23,24]. The antiviral activity of Lf involves binding to SARS-CoV-2 viral particles, hindering spike S attachment to the angiotensin-converting enzyme 2 receptor (ACE-2).

In addition to the significant functions of Lf within the OSS, its roles concerning the posterior segment of the eye warrant special attention. Lf potential impact on retinal health and protection is an emerging area of interest, as evidenced by preliminary preclinical data [25,26]. Results have also been recently reported concerning the suppression of myopia using Lf. In this case, preclinical studies have demonstrated that when administered orally, Lf can exert a profound effect on the progression of lens-induced myopia in mouse models [27,28]. This is achieved through the modulation of extracellular matrix remodeling, implicating the IL-6 and matrix metalloproteinase 2 (MMP-2) axis. Such findings are not just groundbreaking because they showcase Lf systemic impact on ocular structures but also because they offer a non-invasive route to managing myopia, a condition that affects a significant portion of the global population. Nonetheless, oral administration of Lf has to face the issue of degradation by proteolytic enzymes, such as pepsin.

A wide part of this review is focused on Lf in tears since Lf distribution and potential therapeutic implications have been more often linked to tear fluid, rather than to other components (e.g., vitreous humor and retina), which are far less explored. Lf represents one of the most abundant proteins in human tears, locally synthesized by the main lacrimal gland and the corneal and conjunctival epithelial cells [29]. Its level accounts for about 25% of the total tear protein content [30,31], estimated to be between 6 and 8 mg/mL in normal subjects [32]. Tear Lf levels of healthy individuals vary widely according to the literature, although the published data are based on different analytical techniques and poorly characterized subjects. Markedly higher levels than plasma Lf concentrations (1 µg/mL) have been reported, ranging from 1.42 mg/mL by radial immunodiffusion [33] to 2.4 mg/mL by microfluidic technology microchips [34]. It can be concluded that a well-standardized range of tear Lf levels, stratified in population characteristics, is still an unmet need.

There is a general consensus, however, that a significant down-regulation of tear Lf occurs with aging and several pathologies compared to matched control groups. This trend has been shown in patients affected by dry eye disease (DED) [35,36], DED associated with meibomian gland dysfunction [37], keratoconus [38], ocular allergy [39], and contact lens intolerance [40]. Tear Lf lower than 1.1 mg/mL exhibited high diagnostic performance (with a positive likelihood ratio of 4.52) in Sjogren’s syndrome (SS) diagnosis [41], and Lf levels lower than 20% vs. total tear proteins showed a significant association in predicting SS- vs. non-SS-related dry eye (odds ratio 5.5), a much higher value than any other ocular clinical parameter [42].

In this view, tear Lf level determination might be a useful tool for the early diagnosis of several diseases directly “at the eye-side” and offer guidance for clinical decisions and tailored strategies. For this reason, the available methods of quantification of Lf in tear fluid are discussed in detail in Section 2. Considering the protective role that tear Lf exerts on the ocular surface, and its down-regulation by physiological, age-dependent mechanisms or disease onset, the strategy to replenish Lf in tears has been attempted by means of topical treatments with exogenous Lf [43] or dietary assumption [44,45]. The strategies for the ocular delivery of Lf are discussed in Section 3, including both an extensive discussion of the methods concerning the OSS and an overview of possible delivery methods for conditions and diseases of the posterior segment.

## 2. Methods of Quantification of Lf in Tear Fluid

Tear fluid analysis is now considered a source of valuable information about the condition of the eyes and systemic diseases, with the eye being interpretable, like a window to these diseases [20,32,46]. Collection is the first and perhaps the main challenging phase in any analytical procedure on tears, which represent a physiologically low-volume liquid biopsy under normal conditions and are even reduced in several disease states. This pre-analytical step is in fact widely recognized to impact analysis, and standard operating procedures (SOPs) have been proposed [47]. The most used methods to collect samples for Lf detection in tears are uptake by a capillary tube and adsorption by Schirmer strips or polyester wicks [32,35,48].

Various detection methods have been employed and have proven their ability in the quantification of tear Lf, with variable sensitivity and accuracy [49]. These analytical methods are briefly described below and summarized in Table 1.

### 2.1. Radial Immunodiffusion

This is a relatively simple, quantitative method that does not use expensive or hyphenated instruments. It applies the diffusion of an antigen in antibody-conjugated agar gels. The antigen is allowed to diffuse radially through a thin layer of antibody-containing agar and forms a circle of precipitation. Lf from the center conjugates with its antibody and diffuses through the agar; the area of the ring reflects its concentration [33]. This technology was commercialized as the Lactoplate immunoassay test [50], for some time utilized in research and clinical settings, and no longer available.

### 2.2. Enzyme-Linked Immunosorbent Assay (ELISA)

This is a rapid and accurate immunological technique based on the specific reactions of antigens and antibodies and is extensively used for the quantification of proteins, including Lf, with high accuracy [51]. A high number of articles reported the application of ELISA to Lf detection in tears and the method can still be considered as an ideal approach for this purpose. However, the relatively high cost of the reagent kits and the laborious and time-consuming procedure have prevented this technique from being utilized in daily clinical practice [49]. Sandwich immunoassays for Lf detection in milk powder have displayed a limit of detection (LOD) of 3.23 ng/mL [52].

### 2.3. Reversed Phase High–Performance Liquid Chromatography (RP-HPLC)

This is a key instrumental analysis for the separation and characterization of proteins and peptides. Improved RP-HPLC methods for the determination of bovine Lf reached an LOD of 1 μg/mL [53]. The result can be acquired within a few minutes, which makes it a versatile tool in biomedical application [54]. Nonetheless, the method is limited by low specificity. This limitation is overcome by its hyphenation with mass spectrometry (MS).

### 2.4. Mass Spectrometry (MS)

MS-based proteomics has been used, in either targeted or untargeted approaches, for the detection and quantification of Lf in small volumes of human tears without prior purification or fractionation of protein components and bypassing the need for antibodies [55,56]. In a typical bottom-up approach, protein samples are digested by a specific protease, e.g., trypsin, and peptides are separated on a C18 reverse-phase column, hyphenated with an electrospray ionization (ESI) sample source, detected by MS and identified by MS/MS procedures and database search. Relative protein quantitation has been performed by either isotope-based or isotope-free approaches [32]. Quantitative shotgun proteomics, using iTRAQ technology coupled with 2D-nano-LC-nano-ESI-tandem MS (MS/MS), has been used for the identification and relative quantitation of tear proteins, including Lf, leading to a tear protein-based algorithm for DED diagnosis [57].

Targeted, quantitative proteomics is typically performed by multiple reaction monitoring (MRM). Such an approach has not been applied to tear samples yet, but has been successfully applied to Lf quantitation from dairy products [58,59]. By this method, unique peptides are used as a protein fingerprint. These ions are identified by specific precursor–product transitions in the MRM procedure and quantified by the integration of the corresponding chromatographic peak area. Absolute quantitation can be achieved by comparison to an isotopically labeled, synthetic, internal standard. The high specificity of the MRM transitions and the high resolution and accuracy of modern instrumentation make this method a promising approach also for relative and absolute quantitation of Lf from complex biological matrices. These features come along with the highest sensitivity (LOD ~2 × 10^−11^ M) [59].

It is reasonable to expect that quantitative, MS-based techniques will see the fastest development among methods for Lf quantitation in the lacrimal fluid. Indeed, pilot experiments have been successfully performed, as mentioned above. Although expensive, the machinery is already present in almost any analytical facility for other purposes. The sensitivity is compatible with low sample consumption and the pipeline can be optimized to minimize sample manipulation and maximize automation. The versatility of the technique could be exploited for investigating, at the same time, protein post-translational modifications, profiling proteoforms and isoforms, and monitoring exogenous (delivered) Lf.

### 2.5. Electrophoresis–Capillary and SDS-PAGE

Both capillary electrophoresis (CE) and conventional SDS-PAGE have been used to analyze tear Lf [60]. The determination of bovine Lf in infant formula by CE reached an LOD of 5 μg/mL [61]. The CE-based systematic evolution of ligands by exponential enrichment (CE-SELEX) could lower the LOD to 78 ng/mL [62]. More recently, microfluidics-based platforms have been introduced for the quantification of proteins by miniaturized capillary gel electrophoresis, in conjunction with an appropriate LabChip kit. The method is accurate and affordable in terms of cost and time and is used in clinical settings on a routine basis to provide tear electropherogram “fingerprints” [34,63,64,65].

### 2.6. Miscellaneous Methods

A novel and innovative, microfluidic, paper-based analytical device (μPAD) for assessing the tear Lf of DED patients has been reported [66]. The data reflect the severity of the disease, thus helping clinicians in patient management. The device is based on the specific fluorescence emission of terbium upon Lf binding [67] and has an LOD of 110 μg/mL [68]. Competition experiments by “native MS” [69,70] have shown that terbium binds to the iron-binding sites of the Lf paralog transferrin [71].

Nano-based electrochemical or colorimetric sensor-based platforms represent revolutionary analytical methods that could detect Lf in real time and be applied in the clinical routine [72]. The performance of the biosensing system in measuring tear Lf appears to be accurate and has been implemented in fluorescein-based devices [73], electrochemical biosensors [74], and surface plasmon resonance (SPR) spectroscopy [75].

Despite the achievements reported in this and the previous paragraphs, there is still a need for quick, robust, reliable, and cost-effective detection systems for tear Lf (and, in general, for tear analytes). The diagnostic testing of small-volume samples is a relatively young field and offers a wide space for further progress.

In this view, recent advances in Lf detection systems tested on commercial or biological samples have a potential for future, wider applications and are briefly described here, although they have not yet been applied to tear fluid. DNA aptamers are a promising class of molecules for the development of biosensors and can be optimized for binding affinity toward a single or multiple target molecule by selecting from large random libraries, by in vitro systems such as SELEX [76,77]. Aptasensors can be combined with a wide array of readout approaches and offer advantages in production and stability, relative to immunoglobulin counterparts. The bottleneck is the aptamer performance in binding affinity and specificity. Relevant to this regard is the identification of a 72 bp DNA aptamer binding Lf with a K_D_ in the range of 10^−8^ M and selectivity above 97% [78,79]. Complex formation was demonstrated on commercial preparations of human Lf by SPR and electrochemical impedance spectroscopy (EIS) as complementary, label-free, detection systems [78]. The LOD by SPR was ~4 × 10^−9^ M.

Another interesting approach combines dynamic light scattering (DLS) with immuno- and boronate-affinity recognition [80]. This method is still based on immunosensing but overcomes the need for a second antibody for detection, as in conventional sandwich ELISA, as well as labeling by fluorophores. An anti-Lf monoclonal antibody is conjugated to magnetic nanoparticles (NPs) and used to capture Lf from commercial preparations of bovine milk powder. After immunomagnetic separation, bovine serum albumin modified with polyvalent phenylboronic acid (BSA@PBA) is added as a cross-linking agent. The reaction of PBA with the *cis*-diol groups of glycosylated Lf promotes aggregation of the functionalized NPs detectable by DLS. The average hydrodynamic diameter of the aggregates is directly proportional to Lf concentration. The reported LOD of this method is ~1 × 10^−5^ M.

## 3. Strategies for Ocular Delivery of Lf

**Figure 4:**
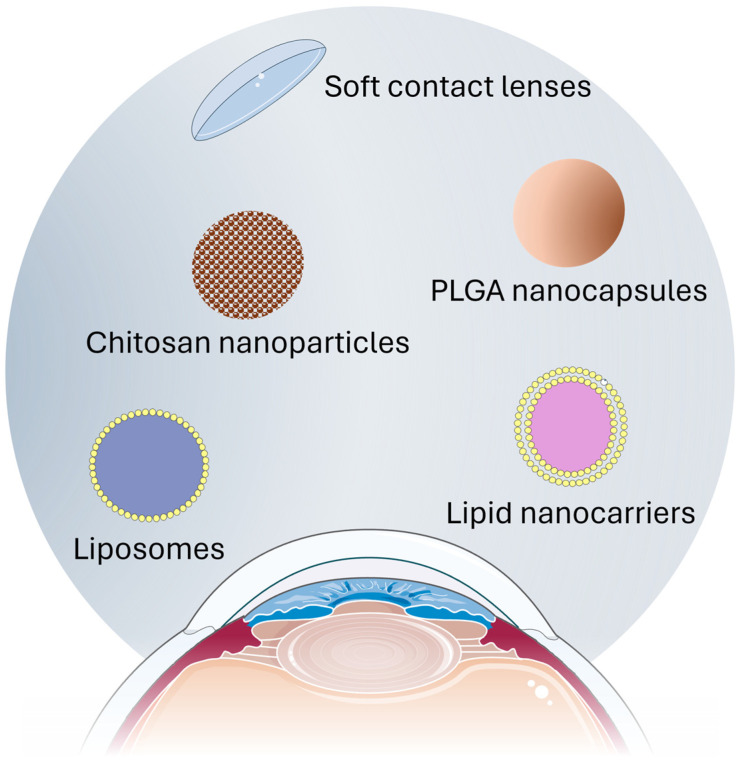
Lf delivery systems for topical ophthalmic administration. PLGA: poly(lactic-co-glycolic) acid.

Topical administration is a widespread approach to treat the OSS as it does not cause pain to the subject and is easy to manage. Nevertheless, ocular therapies exhibit a rapid elimination process via the conjunctiva and nasolacrimal duct, leading to a short pre-corneal half-life of drugs, typically lasting only a few minutes. Consequently, frequent administration becomes necessary [81]. In this view, the use of controlled release strategies, such as nanotechnological carriers, contact lenses, and others, for delivering active principles has garnered significant attention in recent years (Figure 4 and Table 2). These innovative approaches enable the enhanced stability, permeability, and bioavailability of molecules, presenting notable advantages over conventional pharmaceutical forms [82].

### 3.1. Clinical Studies

#### 3.1.1. Topical Administration

Despite the strong interest in clinical applications of Lf, there are just a few comprehensive clinical studies on topical, ophthalmic Lf formulations [92] (Table 2).

One study focused on chronic conjunctivitis [83] and employed Lacto eye drops, which contain Lf as the main active ingredient. After a one-month administration of this formulation, there was a sanitizing effect with no conjunctival microbiota growth and subjective improvement in complaints in all patients. This formulation was effective in increasing tear Lf concentration and normalizing the conjunctival microbiota. The Lacto eye drops demonstrated immune-modulating effects and antimicrobial properties, contributing to the maintenance of ocular health.

A study on post-operative endophthalmitis employed eye drops containing liposomal Lf [91]. The inclusion of Lf into liposomes enhanced protein stability and mitigated the high nasolacrimal duct drainage that may reduce the treatment efficacy.

#### 3.1.2. Oral Administration

Reported clinical studies also involve Lf delivery in contexts other than topical ophthalmic applications, such as oral administration in patients with DED post-cataract surgery [45] or SS [93]. Furthermore, while numerous clinical trials explored the use of Lf in other medical fields like cancer, intestinal diseases, and COVID-19, only one randomized–controlled trial has been reported on the effects of a dietary supplement containing Lf, among other components, on DED [44].

The oral delivery of Lf through advanced nanosystems holds significant promise in biomedical research, addressing challenges related to enzymatic degradation in the gastrointestinal tract [94]. Nanocarriers composed of materials like starch and proteins may undergo hydrolysis by amylases and proteases. In contrast, materials such as pectin and alginate resist enzymatic action and pH changes, being susceptible only to colonic microbiota [95].

Innovative delivery systems, such as NP-based approaches, encapsulate Lf in nanocarriers (liposomes, polymeric NPs, and lipid NPs), providing protection in the stomach and enabling controlled release in the digestive system [94]. Lipid NPs, including solid lipid NPs and nanostructured lipid carriers, show the potential to enhance the oral bioavailability of Lf [95]. Various experimental methodologies, including microparticles, NPs, and liposomes, have been explored to evaluate the impact of different carrier systems on Lf stability. Recent comprehensive reviews have focused on these oral delivery systems, which facilitate sustained release, safeguarding Lf during gastric transit and ensuring efficient absorption in the intestines [94,96,97,98,99,100,101]. Consequently, the oral administration of Lf through advanced delivery systems emerges as a promising avenue of treatment, offering a convenient and patient-friendly therapeutic approach.

### 3.2. Preclinical Studies

While the clinical applications of Lf formulations remain limited, this review underscores the promising potential based on preclinical developments and highlights the need for more focused clinical research in this area. Several preclinical studies apply biocompatible Lf delivery systems for topical ophthalmic administration as a pharmacological alternative for various ocular diseases or discomforts and are briefly discussed in the following paragraphs (Table 2).

The delivery of Lf has been proposed for the first treatment of keratoconus as a pharmacological alternative to invasive surgical interventions [86]. In this view, Lf-loaded contact lenses were suggested as a new and promising therapeutic strategy for the treatment of ocular surface diseases [85,90]. In particular, Lf released in vitro from contact lenses was able to protect human epithelial cells from the effects of oxidative stress. The amount of Lf loaded onto one contact lens was on the order of dozens of micrograms and the release was between 49% and 100% of the adsorbed protein amount, depending on the material. The Lf released from the contact lenses maintained its antioxidant potential for at least 24 h by protecting human epithelial cells from the damaging effects of induced oxidative stress [90]. The antioxidant activity of Lf-loaded contact lenses was also investigated in the case of epithelial cells incubated in tears from patients affected by keratoconus [85]. Both studies demonstrate the in vitro efficacy of Lf-loaded contact lenses in protecting against oxidative stress.

Nanostructured lipid carriers (NLCs) showed a nanocapsular structure with an aqueous core ideal for Lf immobilization [87]. In addition, their mucoadhesive properties led to prolonged residence time and ensured a deeper penetration of therapeutic agents into the corneal layers [87]. An alternative to NLCs is represented by chitosan-based NPs [86]. In vitro release profiles indicated that Lf delivery from the polymeric matrix was enhanced, prolonged, and controlled. These formulations exhibited stability, lacked cytotoxicity issues, and possessed suitable mucoadhesive properties [86]. Both studies underscored the significance of particle size, morphology, and surface charge in influencing the formulation properties for effective ocular drug delivery.

Ocular diseases such as fungal and viral infections, as well as comorbid conditions like DED, are challenging to treat due to natural ocular barriers such as the corneal epithelium and tear fluid that restrict the passage of drugs. In this view, it is necessary to develop new systems for the release of biocompatible Lf for topical ophthalmic administration with the aim of improving the permanence of Lf, i.e., its bioavailability. In addition, traditional treatments, including corticosteroids, antihistamines, and nonsteroidal anti-inflammatory drugs, often cause significant side effects upon long-term use. 

In a recent study, bovine Lf was loaded onto PLGA NPs [88] by using an advanced modified double emulsion technique [102,103]. The NPs were found to be non-toxic and effectively inhibited the inflammatory response triggered by lipopolysaccharides (LPSs). To evaluate how well NP-encapsulated Lf permeated through the eye, isolated corneas from New Zealand rabbits were used. It was observed that Lf from NPs permeated slightly faster than free Lf, suggesting differences in permeability and flux rates. This difference could be ascribed to the higher lipophilic properties of NPs compared to free Lf, given that the epithelial layer of the cornea consists primarily of lipids, which limits the penetration of hydrophilic substances and acts as a barrier to drug delivery within the eye [104]. Additionally, the study compared the molecular permeability of both Lf-loaded nanocapsules and free Lf after 24 h. The healing and protective capabilities of Lf-loaded PLGA NPs were further investigated using an inflammation model induced by arachidonic acid. The treatments were effective, although there was no notable difference in the amount of Lf retained in the corneal region. In vivo studies on rabbits also underscored the potential of these NPs to protect against and heal ocular inflammation. Interestingly, the pharmacokinetic profiles of both free Lf and liposome-encapsulated Lf showed improved parameters over those reported in the previous study by the same team [89].

Other studies focused on the development of slow-release nanocarrier systems. These represent an important target of study and such technologies can increase the Lf bioavailability on the ocular surface, as well as ensure high patient compliance [105].

Another emerging and promising technology is represented by 3D printing, which can support the creation of structures and materials with precise control over their physical properties [106]. Some research has been conducted on the 3D printing of Lf frameworks for potential biomedical applications. For example, Lf-loaded alginate hydrogel scaffolds were successfully 3D printed [107] and exhibited good biocompatibility and supported the growth of human mesenchymal stem cells. Additionally, a 3D-printed Lf-based hydrogel used as a wound medication demonstrated favorable mechanical properties and the ability to release Lf [108].

The previous paragraphs of this section addressed the preclinical studies on keratoconus, fungal and viral infections, and ocular inflammation. Miscellaneous strategies to increase ocular bioavailability are discussed below. Several review articles have been recently published on various drugs and molecules for ocular use besides Lf [109,110,111,112]. These strategies could be borrowed to improve the ocular bioavailability of Lf, as well. In particular, polymers are used to increase the solubility of some drugs or their residence time on the eye by increasing the viscosity of the product. They are also used to increase the penetration depth in the ocular tissues by increasing the drug permeability, improving the patient experience. For example, to increase the solubility of some hydrophobic drugs, water-soluble synthetic polymers, such as PEG and polyvinyl alcohol (PVA), have been used [113,114]. Among polysaccharides, cellulose, its derivative carboxymethylcellulose (CMC), and hyaluronic acid (HA) are also highly hydrophilic [110]. On the contrary, synthetic polymers such as poly(glycolic acid), poly(lactide), poly(lactic-co-glycolic acid), and poly(caprolactone) (PGA, PLA, PLGA, and PCL, respectively) are hydrophobic polyesters and can be used as drug carriers for proteins across, for example, mucus membranes [81,84,115]. They can also be used to control drug release since their in vivo biodegradability varies depending on monomer ratios and end groups [110]. Some works focused on polyester NPs, for example, PCL, embedded in contact lenses [116,117]. In other cases, the molecules of interest were directly incorporated into the contact lens, which represented the delivery vehicle [118,119,120]. Hydroxypropyl methylcellulose (HPMC), guar gum, pullulan, and HA are often used as viscosity enhancers and lubricating agents [110]. Interestingly, polymers like chitosan possess a positive charge [121], which confers mucoadhesive properties. The positive charge also facilitates penetration through the cornea [110] so that these polymers can serve as a coating for anionic drugs with lower biocompatibility.

While the conditions discussed so far primarily concern the ocular surface, it is essential to recognize that the posterior segment of the eye can also be affected by various pathologies that can benefit from Lf administration. For example, the retina and the RPE are critical sites of inflammation associated with debilitating retinal diseases, such as uveitis and age-related macular degeneration (AMD), where Lf protective properties could offer significant therapeutic benefits [122]. Nonetheless, the retina represents a challenging target for drug delivery. Topical administration proves inefficient, as most of the medication is washed away from the ocular surface or absorbed systemically. Additionally, the ocular surface epithelium limits drug penetration. On the other hand, systemic administration is hindered by the BRB [123]. Nano-formulations of Lf are being explored for their potential to deliver therapeutic agents across BRB [105]. Such nano-formulations are particularly promising for conditions like retinitis pigmentosa and AMD, where oxidative stress and inflammation play key roles in disease progression. Given these challenges, there is a crucial need for innovative delivery systems specifically engineered to effectively transport therapeutic agents directly to the target site where they can deliver their beneficial effects.

## 4. Conclusions

The development, optimization, and clinical relevance of Lf-delivery systems specifically tailored for ocular applications are marking significant strides in addressing the unique challenges associated with ocular drug delivery. Lf, a naturally occurring multifunctional protein, exhibits potent antimicrobial, anti-inflammatory, and immunomodulatory properties, making it an attractive candidate for treatment, prevention, and diagnosis for a wide range of ocular conditions.

Lf therapeutic potential spans a wide range of ocular conditions, demonstrating its versatility and broad applicability in eye health. The literature reports benefits of Lf in various diseases and conditions, which can be categorized based on the anatomical regions of the eye they affect. Indeed, Lf has shown promise in treating both OSS conditions, such as DED, keratoconus, and infections, and posterior segment diseases, including AMD and uveitis. Notably, Lf has proven to be beneficial also in myopia management.

However, the delicate and complex nature of the ocular environment necessitates innovative delivery strategies to overcome barriers to effective treatment. Nanotechnology-based delivery systems, including NPs, liposomes, and hydrogels, have shown particular efficacy in enhancing the bioavailability, stability, and sustained release of Lf. The choice between these delivery systems should take into account the specific requirements of the therapeutic application, including the nature of the drug, the required duration of therapy, and the target site. However, for sensitive and biologically active molecules such as Lf, the protective, biocompatible, and versatile nature of liposomes and lipid NPs often makes them particularly suitable candidates for ocular drug delivery.

Beyond its extensively documented antibacterial activity, Lf ability to cross the BRB and participate in cell cycle control offers unique advantages over other transferrin proteins, highlighting its potential as a versatile carrier for drug delivery. Moreover, the emerging role of Lf in treating and preventing ocular manifestations of systemic infections, such as conjunctivitis associated with SARS-CoV-2 infection, underscores its capability not only to chelate iron but also to interact directly with pathogens while exerting immunomodulatory effects. Future randomized, controlled studies will be crucial to further elucidate and confirm the efficacy of Lf in a broader spectrum of ocular conditions, ensuring that this natural protein can be effectively utilized in clinical settings to improve patient outcomes in eye health.

In this framework, the need for multidisciplinary approaches is evident, combining insights from biochemistry, pharmacology, material science, and nanotechnology. In emphasizing the significance of cross-disciplinary innovation, it is crucial to acknowledge the potential for advancements in ocular delivery systems by drawing inspiration from technologies developed for other pathologies. The exchange of knowledge and methodologies across different fields of medical research has historically accelerated the development of novel therapeutic strategies, and this approach remains highly relevant in the context of improving Lf delivery within the eye.

Technologies that have revolutionized drug delivery in oncology, neurology, and endocrinology offer valuable lessons for ocular pharmacotherapy. For instance, the precise targeting mechanisms employed in cancer therapy to deliver chemotherapeutic agents directly to tumor cells, while minimizing systemic toxicity, can inspire similar strategies for targeting specific cells or tissues within the eye. This could enhance the efficacy of Lf delivery to areas most affected by the disease, such as the retina in the case of AMD or the cornea and conjunctiva in the case of DED and infections.

Moreover, the progress made in developing responsive drug delivery systems that can release the loaded compound in response to specific physiological stimuli (e.g., changes in pH, temperature, or enzymatic activity) could be adapted to create smart ocular delivery systems. These systems could potentially release Lf in response to the onset of inflammation or infection, providing timely and localized therapy that minimizes the need for frequent dosing and reduces the risk of side effects.

## Figures and Tables

**Table 1:** Detection methods commonly employed for Lf detection and quantitation. ELISA: enzyme-linked immunosorbent assay; HPLC: high-performance liquid chromatography; MS: mass spectrometry; RP: reverse-phase; SDS-PAGE: sodium dodecyl sulfate polyacrylamide gel electrophoresis.

Detection Method	Principle	Highlights
Radial immunodiffusion	Antigen (Lf) diffuses radially from a well into a gel containing specific antibodies, forming a precipitin ring whose diameter is proportional to Lf concentration	Simple High specificity Requires long incubation time (24–48 h)
ELISA	Antigen (Lf) binds to an antibody coated on a plate. A secondary enzyme-linked antibody binds the antigen, and a substrate is added to produce a measurable change	High sensitivity and specificity Relatively quick Suitable for high-throughput screening
RP-HPLC	Separation of proteins based on their hydrophobicity using an RP column and detection by UV or MS	High resolution and sensitivity Suitable for complex mixtures Requires method development and optimization
MS	Ionization, separation of ions based on their mass-to-charge ratio (*m*/*z*), and detection to generate a mass spectrum for quantification	High specificity and sensitivity Can provide absolute quantitation Allows detection of post-translational modifications
Capillary electrophoresis	Separation of Lf based on its charge-to-size ratio in an electric field within a capillary tube, with detection usually by UV or laser-induced fluorescence	High resolution and efficiency Requires small sample volumes Rapid analysis time
SDS-PAGE	Denaturation and separation of proteins by size in a polyacrylamide gel, followed by staining and densitometry for quantification	Simple Good for determining protein purity Limited quantitative accuracy compared to other methods

**Table 2:** Examples of Lf formulations to treat ocular surface conditions. NLC: nanostructured lipid carrier; NP: nanoparticle; PLGA: poly(lactic-co-glycolic) acid; PEG: poly(ethylene glycol); DED: dry eye disease.

Target Condition	Formulation	Reference
Chronic conjunctivitis	Lf-containing eye drops	[83]
Different ocular syndromes	PLGA-NPs	[84]
Keratoconus	Lf-loaded contact lenses	[85]
	Chitosan/tripolyphosphate and chitosan/sulfobutylether-β-cyclodextrin NPs	[86]
	NLCs	[87]
Ocular inflammation; DED	PLGA-NPs	[88]
	PEGylated PLGA nanospheres	[81]
	Hyaluronic acid-coated liposomes	[89]
Oxidative stress conditions	Lf-loaded contact lenses	[90]
Postoperative endophthalmitis	Liposomal Lf-based eye drops	[91]
